# Supplementary material for: Music and mood regulation during the early stages of the COVID-19 pandemic
Source: PLoS One. 2021 Oct 20;16(10):e0258027. doi: 10.1371/journal.pone.0258027 (PMC8528311; doi:10.1371/journal.pone.0258027)

# S1 Figure. Pairwise Pearson correlations for all behavioral measures of interest and covariates.


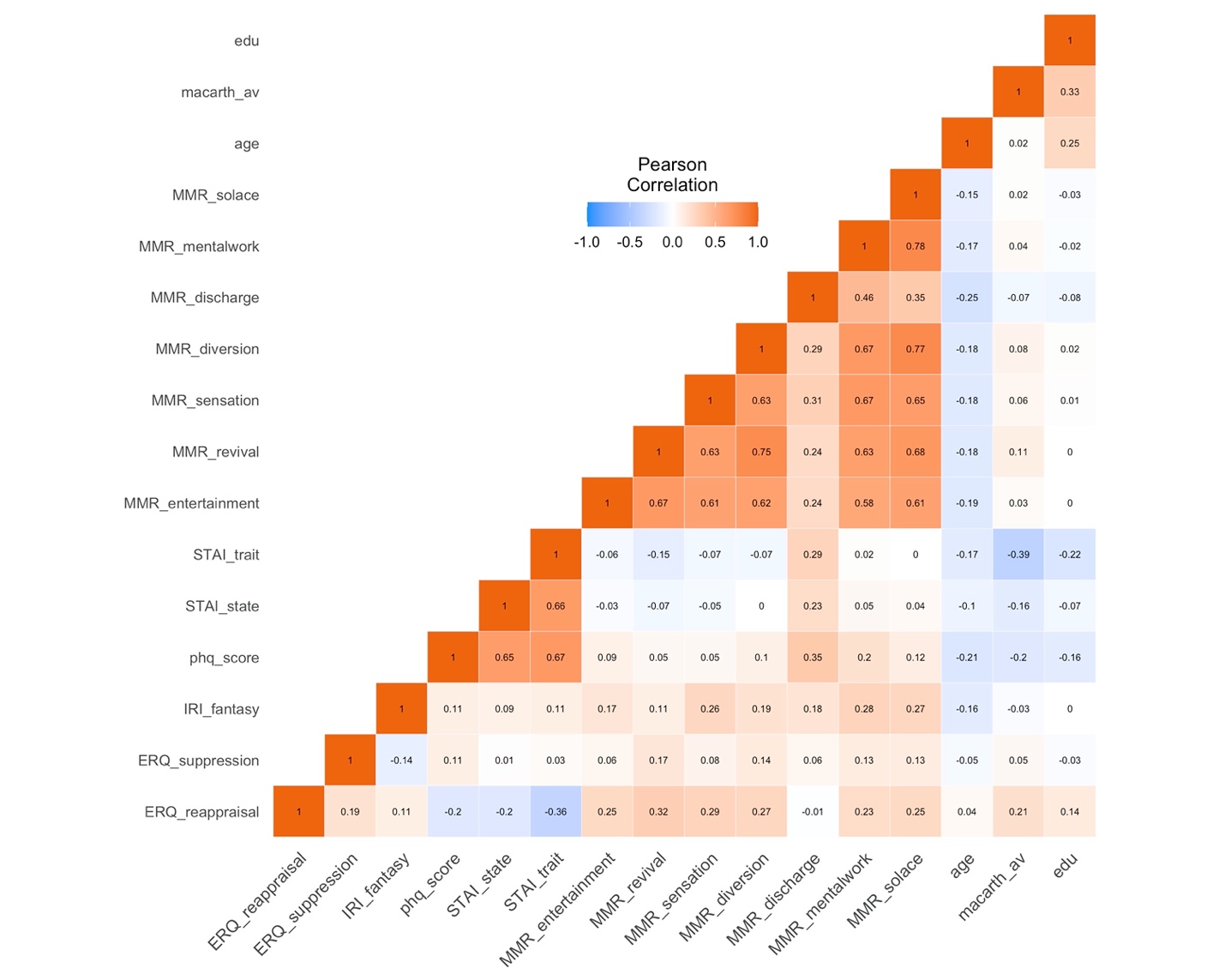

Supplement: S1 Fig — (DOCX) [file pone.0258027.s001.docx]
