# Supplementary figures and images for: Music and mood regulation during the early stages of the COVID-19 pandemic
Source: PLoS One. 2021 Oct 20;16(10):e0258027. doi: 10.1371/journal.pone.0258027 (PMC8528311; doi:10.1371/journal.pone.0258027)

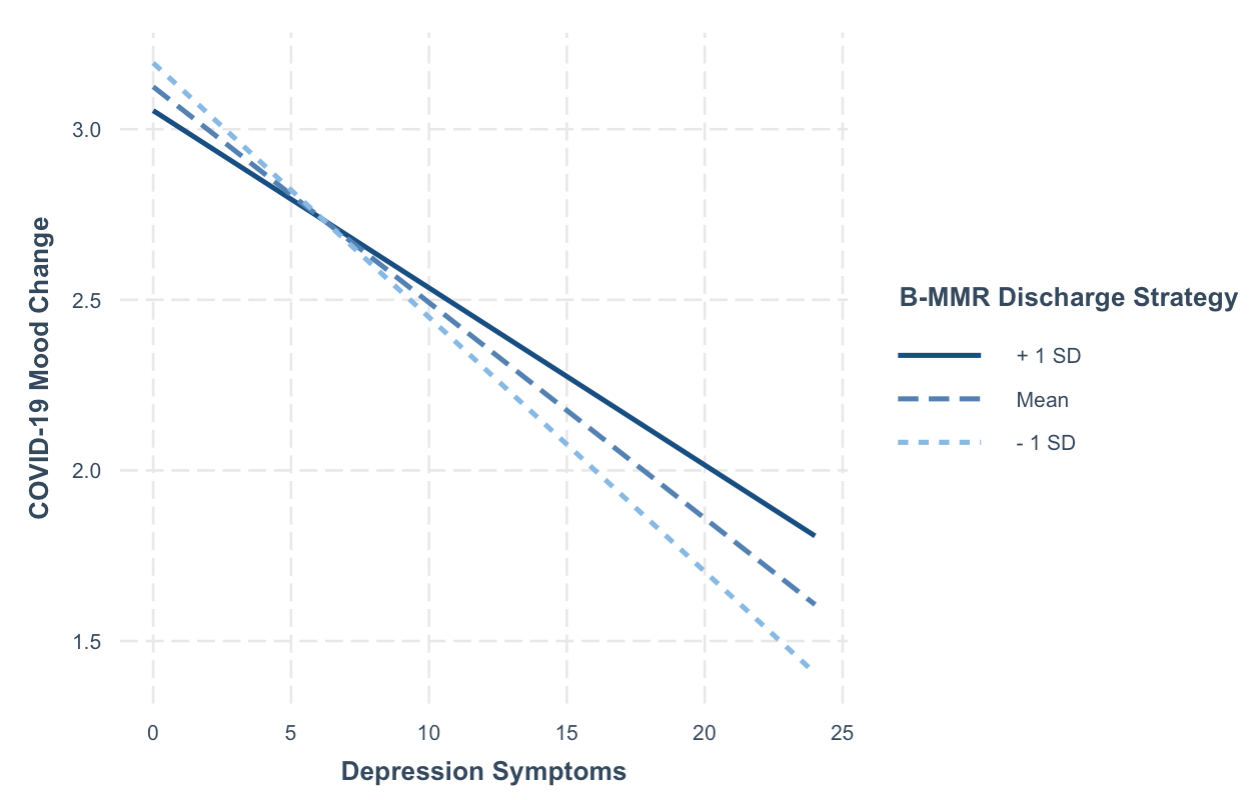
**S2 Figure. Interaction between PHQ-9 and B-MMR Discharge on COVID-19 Mood Change.**

Supplement: S2 Fig — (DOCX) [file pone.0258027.s002.docx]
