# Supplementary material for: Music and mood regulation during the early stages of the COVID-19 pandemic
Source: PLoS One. 2021 Oct 20;16(10):e0258027. doi: 10.1371/journal.pone.0258027 (PMC8528311; doi:10.1371/journal.pone.0258027)
Supplement: S1 Table — (DOCX) [file pone.0258027.s003.docx]

**S1 Table. ANCOVA results, country predicting each Brief Music and Mood Regulation strategy.**

|  | Sum  of  Squares | *df* | Mean  Square | *F* | *p* | η^2^ |
| --- | --- | --- | --- | --- | --- | --- |

*MMR Discharge*

| (Intercept) | 3378.58 | 1 | 3378.58 | 316.84 | .000 |  |
| --- | --- | --- | --- | --- | --- | --- |
| age | 259.37 | 1 | 259.37 | 24.32 | .000^***^ | .06 |
| education | 0.07 | 1 | 0.07 | 0.01 | .934 | .00 |
| gender | 80.46 | 1 | 80.46 | 7.55 | .006^**^ | .01 |
| SES | 48.10 | 1 | 48.10 | 4.51 | .034 | .00 |
| musician | 120.39 | 1 | 120.39 | 11.29 | .001^**^ | .02 |
| country | 74.32 | 3 | 24.77 | 2.32 | .074 | .01 |
| Error | 5811.57 | 545 | 10.66 |  |  |  |

*MMR Diversion*

| (Intercept) | 3136.86 | 1 | 3136.86 | 424.07 | .000 |  |
| --- | --- | --- | --- | --- | --- | --- |
| age | 144.80 | 1 | 144.80 | 19.58 | .000^***^ | .03 |
| education | 0.12 | 1 | 0.12 | 0.02 | .900 | .00 |
| gender | 5.13 | 1 | 5.13 | 0.69 | .405 | .00 |
| SES | 0.93 | 1 | 0.93 | 0.13 | .723 | .00 |
| musician | 35.36 | 1 | 35.36 | 4.78 | .029 | .01 |
| country | 220.27 | 3 | 73.42 | 9.93 | .000^***^ | .05 |
| Error | 4031.40 | 545 | 7.40 |  |  |  |

*MMR Entertainment*

| (Intercept) | 3658.52 | 1 | 3658.52 | 515.85 | .000 |  |
| --- | --- | --- | --- | --- | --- | --- |
| age | 125.64 | 1 | 125.64 | 17.72 | .000^***^ | .04 |
| education | 0.54 | 1 | 0.54 | 0.08 | .783 | .00 |
| gender | 16.02 | 1 | 16.02 | 2.26 | .133 | .00 |
| SES | 2.14 | 1 | 2.14 | 0.30 | .583 | .00 |
| musician | 28.36 | 1 | 28.36 | 4.00 | .046^*^ | .01 |
| country | 51.22 | 3 | 17.07 | 2.41 | .066 | .01 |
| Error | 3865.26 | 545 | 7.09 |  |  |  |

*MMR Mental Work*

| (Intercept) | 3193.43 | 1 | 3193.43 | 486.49 | .000 |  |
| --- | --- | --- | --- | --- | --- | --- |
| age | 73.06 | 1 | 73.06 | 11.13 | .001^**^ | .03 |
| education | 2.66 | 1 | 2.66 | 0.41 | .524 | .00 |
| gender | 11.83 | 1 | 11.83 | 1.80 | .180 | .00 |
| SES | 3.99 | 1 | 3.99 | 0.61 | .436 | .00 |
| musician | 57.69 | 1 | 57.69 | 8.79 | .003^**^ | .02 |
| country | 127.70 | 3 | 42.57 | 6.48 | .000^***^ | .03 |
| Error | 3577.47 | 545 | 6.56 |  |  |  |

*MMR Revival*

| (Intercept) | 2946.89 | 1 | 2946.89 | 383.75 | .000 |  |
| --- | --- | --- | --- | --- | --- | --- |
| age | 134.19 | 1 | 134.19 | 17.47 | .000^***^ | .03 |
| education | 2.01 | 1 | 2.01 | 0.26 | .609 | .00 |
| gender | 5.24 | 1 | 5.24 | 0.68 | .409 | .01 |
| SES | 0.93 | 1 | 0.93 | 0.12 | .728 | .01 |
| musician | 25.51 | 1 | 25.51 | 3.32 | .069 | .01 |
| country | 197.08 | 3 | 65.69 | 8.55 | .000^***^ | .04 |
| Error | 4185.20 | 545 | 7.68 |  |  |  |

*MMR Sensation*

| (Intercept) | 3431.10 | 1 | 3431.10 | 570.04 | .000 |  |
| --- | --- | --- | --- | --- | --- | --- |
| age | 46.23 | 1 | 46.23 | 7.68 | .006^**^ | .03 |
| education | 1.88 | 1 | 1.88 | 0.31 | .577 | .00 |
| gender | 4.99 | 1 | 4.99 | 0.83 | .363 | .00 |
| SES | 1.11 | 1 | 1.11 | 0.18 | .668 | .00 |
| musician | 110.66 | 1 | 110.66 | 18.38 | .000^***^ | .04 |
| country | 127.01 | 3 | 42.34 | 7.03 | .000^***^ | .03 |
| Error | 3280.38 | 545 | 6.02 |  |  |  |

*MMR Solace*

| (Intercept) | 3342.79 | 1 | 3342.79 | 481.94 | .000 |  |
| --- | --- | --- | --- | --- | --- | --- |
| age | 66.31 | 1 | 66.31 | 9.56 | .002^**^ | .02 |
| education | 4.68 | 1 | 4.68 | 0.67 | .412 | .00 |
| gender | 19.01 | 1 | 19.01 | 2.74 | .098 | .01 |
| SES | 8.06 | 1 | 8.06 | 1.16 | .282 | .00 |
| musician | 33.60 | 1 | 33.60 | 4.84 | .028^*^ | .01 |
| country | 168.66 | 3 | 56.22 | 8.11 | .000^***^ | .04 |
| Error | 3780.14 | 545 | 6.94 |  |  |  |
