# Supplementary material for: Music and mood regulation during the early stages of the COVID-19 pandemic
Source: PLoS One. 2021 Oct 20;16(10):e0258027. doi: 10.1371/journal.pone.0258027 (PMC8528311; doi:10.1371/journal.pone.0258027)
Supplement: S1 Text — (DOCX) [file pone.0258027.s004.docx]

**S1 Text. Supporting Methods and Results.**

**S1A Text. Supporting Methods**

**COVID-19 questionnaire to assess distress related to COVID-19 pandemic**

Seven YES/NO questions were asked to assess personal COVID-19 severity. This included the following questions:

1. Do you personally know anyone that tested positive for COVID-19?
2. Do you personally know anyone that has been hospitalized due to COVID-19?
3. Do you personally know anyone that has passed away dues to COVID-19?
4. Have you made any changes to your daily lifestyle due to COVID-19?
5. Are you experiencing a financial loss due to COVID-19?
6. Is the area where you live under mandatory, “stay at home”, “shelter in place” order?
7. How much are you self-isolating (from 100% to None)

The COVID-19 severity portion also included self-assessment of perceived risk and personal distress. These included the following questions:

1. Please rate the overall level of risk to you personally that you perceive from the COVID-19 pandemic (from *1 - none* to *5 - extreme*)
2. How much of a threat do you think COVID-19 (coronavirus) is to the world? (from *1 - none* to *5 - extreme*)

We also asked for change in mood as a result of COVID:

1. In the past 4 weeks have you experienced any change in your mood? (from *1 - significantly worse* to *5 - significantly better*)
2. In the past 4 weeks has your level of general anxiety changed? (from *1 - significantly worse* to *5 - significantly better*)

**S1B Text**. **Supporting Results**

**Responses to COVID-19 by country.**

***COVID-19 severity and perceived risk.***

A significant effect of country was observed in COVID-19 severity (F(3, 546) = 19.75, p < 0.0001, η^2^= 0.09), where participants from Italy reported higher severity than those from the United Kingdom (p < 0.0001) and the United States (p < 0.0001). Participants from India reported higher severity than those from the United Kingdom (p < 0.001), and the United States (p < 0.0001). No differences were observed between countries in perceived personal risk of COVID-19 (p > 0.05).

***Symptoms of anxiety and depression by country.***

No differences were observed between countries in symptoms of depression (PHQ-9; p > 0.05). There was a significant effect of country on state anxiety (F(3, 546) = 4.50, p < 0.05, η^2^= 0.02), where participants from Italy had greater state anxiety than those from the United States (p < 0.05). Differences between countries on trait anxiety were not significant after correcting for multiple comparisons (p > 0.05).

***Mood change by country*.**

A significant effect of country was observed on the use of music to improve mood (F(3, 545 = 17.89, p < 0.0001, η^2^= 0.09), where participants from India used music more to improve mood than did participants from Italy (p < 0.001), the United Kingdom (p < 0.001), and the United States (p < 0.001).

**Music and mood regulation by COVID severity.**

We tested the effects of COVID-19 personal risk in terms of the ways in which music is used to regulate mood. To this end, a series of separate 1-way ANCOVAs were conducted to assess the effects of COVID-19 personal risk on subscales, and overall scores, on the B-MMR while controlling for age, gender, education, SES, and musicianship.

Across the entire dataset, a significant effect of perceived personal risk of COVID-19 was observed on B-MMR total scores (F(1, 544) = 10.84, p < 0.01, η^2^= 0.02), where personal risk of COVID-19 was positively correlated with use of music for mood regulation (𝛃 = 0.13). Personal COVID-19 risk was also significantly positively associated with the Diversion (F(1, 544) =18.34, 𝛃 = 0.18, p < 0.0001, η^2^= 0.03), Entertainment (F(1, 544) = 6.53, 𝛃 = 0.10, p < 0.05, η^2^= 0.01), Mental Work (F(1, 544) = 6.51, 𝛃 = 0.18, p < 0.05, η^2^= 0.01), Revival (F(1, 544) = 5.72, 𝛃 = 0.10, p < 0.05, η^2^= 0.01), and Solace (F(1, 544) = 6.84, 𝛃 = 0.11, p < 0.01, η^2^= 0.01) B-MMR subscales. No other subscales were significantly predicted by perceived COVID-19 risk after FDR correction (ps > 0.05). Interestingly, the degree to which a person was actually affected by the pandemic (COVID-19 severity) was positivity associated with multiple B-MMR subscales, including Revival (F(1, 544) = 8.23, 𝛃 = 0.13, p < 0.01, η^2^= 0.01), Sensation (F(1, 544) = 9.58, 𝛃 = 0.14, p < 0.01, η^2^= 0.02), Solace (F(1, 544) = 9.21, 𝛃 = 0.14, p < 0.01, η^2^= 0.02), and Mental Work (F(1, 544) = 10.30, 𝛃 = 0.14, p < 0.01, η^2^= 0.02), as well as total B-MMR (F(1, 544) = 8.50, 𝛃 = 0.13, p < 0.01, η^2^ = 0.01).

**Additional predictors of subscales of the Brief Music in Mood Regulation Scale**

The trait component of the STAI additionally predicted the use of B-MMR strategies of Revival (F(1, 544) = 9.57, p < 0.01, η^2^= 0.02), and Discharge (F(1, 544) = 31.48, p < 0.0001, η^2^= 0.05), where higher trait anxiety was negatively associated with use of Revival as a strategy (𝛃 = -0.15) and positively associated with use of Discharge (𝛃 = 0.25). ERQ Suppression scores significantly predicted use of the B-MMR Revival strategy, but this was not significant after FDR correction (F(1, 544) = 4.32, 𝛃 = 0.09, p < 0.05, p-adjust = 0.065, η^2^= 0.01). ERQ Suppression did not predict use of any other B-MMR strategy (p > 0.05). ERQ Reappraisal scores significantly predicted the use of total B-MMR (F(1, 544) = 42.08, 𝛃 = 0.26, p < 0.0001, η^2^= 0.06). ERQ Reappraisal scores also significantly predicted use of B-MMR Solace strategy (F(1, 544) = 29.92, 𝛃 = 0.23, p < 0.0001, η^2^= 0.05), Revival (F(1, 544) = 53.45, 𝛃 = 0.30, p < 0.0001, η^2^= 0.08), Entertainment (F(1, 544) = 33.08, 𝛃 = 0.24, p < 0.0001, η^2^= 0.05), Sensation (F(1, 544) = 42.74, 𝛃 = 0.26, p < 0.0001, η^2^= 0.07), Mental Work (F(1, 544) = 23.24, 𝛃 = 0.20, p < 0.0001, η^2^= 0.04).

**Predictors of COVID Mood Change**

We tested whether changes in mood due to COVID-19 were predicted by depression symptoms, or the Discharge strategy of the B-MMR. Increased symptoms of depression (PHQ-9) was associated with more negative mood change due to the COVID-19 pandemic (F(1, 544) = 120.59, 𝛃 = -0.44, p < 0.0001, η^2^= 0.17). A significant interaction was observed between PHQ and Discharge on COVID-19 mood change (F (1, 542) = 5.02, p < 0.05, η^2^ < 0.01) where the relationship between PHQ and negative mood during the pandemic was weakened by higher use of B-MMR Discharge strategy (see S2 Figure). This indicated that, even though increased depression was associated with feeling worse during COVID, the relationship was weaker in people who regulate their mood with music through the discharge strategy.
