# Supplementary material for: Music and mood regulation during the early stages of the COVID-19 pandemic
Source: PLoS One. 2021 Oct 20;16(10):e0258027. doi: 10.1371/journal.pone.0258027 (PMC8528311; doi:10.1371/journal.pone.0258027)
Supplement: S2 Text — (DOCX) [file pone.0258027.s005.docx]

**S2 Text. Additional playlist analyses**

***Reminiscence bump***

Music that evokes autobiographical memories has been shown to induce positive emotions ^1^ and to mediate the relationship between emotion regulation strategy and mental health ^2^. Memories evoked by music are most strongly tied to songs from an individual’s “reminiscence bump” ^3^ defined broadly as a 10-year period in the mid-to late adolescent years ^4^, characterized by rapid growth of social bonds and development of individual preferences. Engaging with feelings of nostalgia, for example through memory-evoking songs, may protect against feelings of loneliness and enforce a sense of personal meaning by reminding the listener of periods of social connectedness and identify formation ^5,6^. In the presence of a pandemic characterized by social isolation, it is possible that music listening habits may shift to include more memory-evoking content.

We were therefore interested to know if songs listened to pre- or post-COVID were more likely to fall within a participant’s reminiscence bump if it was released when the participant was between the ages of 9 and 18. Reminiscence bump song percentage was calculated as the proportion of listed songs within this age period to songs outside of this age period. This was calculated for pre- and post-COVID songs separately, for Italy, the United Kingdom, and the United States. A repeated-measures ANOVA was conducted for reminiscent bump song percentage, with country as the between-subjects factor, and time as the within-subjects factor. No significant effect of time, country, or time x country interaction was observed on reminiscence bump song percentage (p > 0.05).

***Genre***

Chi-square test of association was conducted to assess differences in musical genre from pre- to post-COVID***.*** There was a significant association between music genre and time (*X*^2^ (16, *N* = 480) = 416, *p* < 0.0001). Participants listened to classical (p < 0.001), jazz (p < 0.0001), Latin, rock (p < 0.001) music more pre-COVID-19 than post-COVID-19. Participants listened to country (p < 0.0001), folk (p < 0.05), hip-hop (p < 0.01), K-pop (p < 0.0001), musical theater (p < 0.001), pop (p < 0.01), and trap (p < 0.0001) music post-COVID-19 than pre-COVID-19.

References

1. Maksimainen, J., Wikgren, J., Eerola, T. & Saarikallio, S. The Effect of Memory in Inducing Pleasant Emotions with Musical and Pictorial Stimuli. *Sci. Rep.* **8**, 1–12 (2018).

2. Blais-Rochette, C. & Miranda, D. Music-evoked autobiographical memories, emotion regulation, time perspective, and mental health. *Music. Sci.* **20**, 26–52 (2016).

3. Janata, P., Tomic, S. T. & Rakowski, S. K. Characterisation of music-evoked autobiographical memories. *Memory* **15**, 845–860 (2007).

4. Jansari, A. & Parkin, A. J. Things that go bump in your life: Explaining the reminiscence bump in autobiographical memory. *Psychol. Aging* **11**, 85–91 (1996).

5. Sedikides, C., Wildschut, T., Arndt, J. & Routledge, C. Nostalgia. *Curr. Dir. Psychol. Sci.* **17**, 304–307 (2008).

6. Wildschut, T., Sedikides, C., Arndt, J. & Routledge, C. Nostalgia: Content, triggers, functions. *J. Pers. Soc. Psychol.* **91**, 975–993 (2006).
