# Supplementary material for: Music and mood regulation during the early stages of the COVID-19 pandemic
Source: PLoS One. 2021 Oct 20;16(10):e0258027. doi: 10.1371/journal.pone.0258027 (PMC8528311; doi:10.1371/journal.pone.0258027)
Supplement: Attachment — Submitted filename: Response to reviewer.docx [file pone.0258027.s006.docx]

Dear Dr. Koelsch,

Thank you and the reviewer for the helpful reviews on our manuscript. We wish to express our appreciation for reviewer’s acknowledgement of our work and for their suggestions and correction, which have improved the manuscript. We have revised the text according to their suggestions and queries. Below, we detail the changes point by point to the reviewer’s comments. Enclosed, please see the revised version of the manuscript.

Sincerely,

Sarah Hennessy, Matthew Sachs, Jonas Kaplan, and Assal Habibi

**1. Please ensure that your manuscript meets PLOS ONE's style requirements, including those for file naming. The PLOS ONE style templates can be found at**

[**https://journals.plos.org/plosone/s/file?id=wjVg/PLOSOne_formatting_sample_main_body.pdf**](https://urldefense.com/v3/__https://journals.plos.org/plosone/s/file?id=wjVg*PLOSOne_formatting_sample_main_body.pdf__;Lw!!LIr3w8kk_Xxm!6dO-FmlVklDQJSgje7ZvxiGOMd4PBx6yXOf38H_p_VasD_2k5fjQP_vSMEiqyg$) **and**

[**https://journals.plos.org/plosone/s/file?id=ba62/PLOSOne_formatting_sample_title_authors_affiliations.pdf**](https://urldefense.com/v3/__https://journals.plos.org/plosone/s/file?id=ba62*PLOSOne_formatting_sample_title_authors_affiliations.pdf__;Lw!!LIr3w8kk_Xxm!6dO-FmlVklDQJSgje7ZvxiGOMd4PBx6yXOf38H_p_VasD_2k5fjQP_s1ppTZuQ$)

2. Thank you for including your ethics statement: "This study and all protocols were approved by the University of Southern California Institutional Review Board (UP-20-00271). Data were collected and analyzed anonymously. "

a) Please provide additional details regarding participant consent. In the ethics statement in the Methods and online submission information, please ensure that you have specified **(**1) whether consent was informed and (2) what type you obtained (for instance, written or verbal, and if verbal, how it was documented and witnessed). If your study included minors, state whether you obtained consent from parents or guardians. If the need for consent was waived by the ethics committee, please include this information. If you are reporting a retrospective study of medical records or archived samples, please ensure that you have discussed whether all data were fully anonymized before you accessed them and/or whether the IRB or ethics committee waived the requirement for informed consent. If patients provided informed written consent to have data from their medical records used in research, please include this information.

Once you have amended this/these statement(s) in the Methods section of the manuscript, please add the same text to the “Ethics Statement” field of the submission form (via “Edit Submission”).For additional information about PLOS ONE ethical requirements for human subjects research, please refer to [http://journals.plos.org/plosone/s/submission-guidelines#loc-human-subjects-research](https://urldefense.com/v3/__http://journals.plos.org/plosone/s/submission-guidelines*loc-human-subjects-research__;Iw!!LIr3w8kk_Xxm!6dO-FmlVklDQJSgje7ZvxiGOMd4PBx6yXOf38H_p_VasD_2k5fjQP_vNi4ODtA$).

This information has been added to lines 145-148 and will be amended in the ethics statement on the online submission.

**3.** We note that the grant information you provided in the ‘Funding Information’ and ‘Financial Disclosure’ sections do not match.

When you resubmit, please ensure that you provide the correct grant numbers for the awards you received for your study in the ‘Funding Information’ section.

We have corrected this inconsistency.

4. Thank you for stating the following in the Acknowledgments Section of your manuscript:

“The Brain and Music Program at the Brain and Creativity Institute is supported by the GRoW at Annenberg Foundation, the Los Angeles Philharmonic Association and the Van Otterloo Family Foundation”

We note that you have provided additional information within the Acknowledgements Section that is not currently declared in your Funding Statement. Please note that funding information should not appear in the Acknowledgments section or other areas of your manuscript. We will only publish funding information present in the Funding Statement section of the online submission form.

Please remove any funding-related text from the manuscript and let us know how you would like to update your Funding Statement. Currently, your Funding Statement reads as follows:

“The Brain and Music Program (AH) at the Brain and Creativity Institute is supported by the GRoW at Annenberg Foundation ([https://growannenberg.org/](https://urldefense.com/v3/__https://growannenberg.org/__;!!LIr3w8kk_Xxm!6dO-FmlVklDQJSgje7ZvxiGOMd4PBx6yXOf38H_p_VasD_2k5fjQP_tFusoNhA$)), the Los Angeles Philharmonic Association ([https://www.laphil.com/](https://urldefense.com/v3/__https://www.laphil.com/__;!!LIr3w8kk_Xxm!6dO-FmlVklDQJSgje7ZvxiGOMd4PBx6yXOf38H_p_VasD_2k5fjQP_tjmoXB5Q$)) and the Van Otterloo Family Foundation. The funders had no role in study design, data collection and analysis, decision to publish, or preparation of the manuscript.”

Please include your amended statements within your cover letter; we will change the online submission form on your behalf.

We have removed any funding-related text from the manuscript and have included funding statement in the cover letter.

5. Please include your full ethics statement in the ‘Methods’ section of your manuscript file. In your statement, please include the full name of the IRB or ethics committee who approved or waived your study, as well as whether or not you obtained informed written or verbal consent. If consent was waived for your study, please include this information in your statement as well.

We have included the full ethics statement in the Methods section on lines 145-148.

6. Please include captions for your Supporting Information files at the end of your manuscript, and update any in-text citations to match accordingly. Please see our Supporting Information guidelines for more information: [http://journals.plos.org/plosone/s/supporting-information](https://urldefense.com/v3/__http://journals.plos.org/plosone/s/supporting-information__;!!LIr3w8kk_Xxm!6dO-FmlVklDQJSgje7ZvxiGOMd4PBx6yXOf38H_p_VasD_2k5fjQP_vseRpBGA$).

We have reformatted our supporting information files, added appropriate captions, and updated all in-text citations.

Reviewers' comments:

Reviewer's Responses to Questions

Comments to the Author

Reviewer #1: The study considers an interesting topic and the writing style of the paper is good. However the paper needs considerable work to make it ready for publication. In particular I would recommend that the hypotheses to be tested be narrowed, particularly to focus on the findings relevant to music use in COVID, allowing the statistical analyses performed to also be more focused. The focus should be well justified in the introduction. At the moment the hypotheses as somewhat vague and the statistical tests are therefore numerous and rather exploratory in nature. More specific comments are below.

We thank the reviewers for this helpful comment. In the revised manuscript, we have reorganized and clarified our hypotheses (lines 129-141) at the onset and the specific analyses/statistical tests that we used to address these hypotheses. We do want to acknowledge that given the nature of the topic (how people were using music for mood regulation during the early stages of the COVID-19 pandemic), we prioritized the importance of collecting data quickly and relatively broadly, so as to not miss out on this opportunity to study such an important, world-wide stressful event. Because of this, we did not have clear hypotheses about every measure that was included in the original survey. We try to clarify in the revised manuscript which measures were used to test specific hypotheses and which were exploratory.

p.3 – Reference needed to support the definition provided for “affect regulation”

We have added this reference on line 68.

p. 4 – Mechanisms by which music listening can alter affective states – this is by no means an exhaustive list and more literature could be cited here in order to provide a more comprehensive examination of the topic. E.g. plenty of research indicates that music can also alter affective states by reconnecting people with memories or via messages of hope in the lyrics, or can re-energise via processes of entrainment, it can also provide a sense of social connection and comfort. It is also worth noting the potential for music to cause negative alterations to affective states.

We have added elaboration of the mechanisms by which music listening can alter affective states on lines 57, 59-62. We have also acknowledged the potential for music to cause negative alterations to affective states on lines 62-64.

Also the B-MMR was apparently developed in 2012, which does not seem too recent.

We have amended this sentence at line 69 to note the year of development.

A more recent scale was developed by the same author as the B-MMR, i.e. the HUMS (Healthy & Unhealthy Uses of Music Scale), which might be useful to note here too.

We have now mentioned the HUMS in the introduction at line 64. We did not use the HUMS in this particular study, as this scale was developed to assess “music engagement of young people as an indicator of healthy/unhealthy behavior, from the particular perspective of proneness for depression”. Given that the goal of this investigation was to examine the potential role of music to regulate emotions in a variety of ways in adults, rather than assessing the presence of unhealthy behaviors that might indicate depression proneness in children and adolescents, we felt the BMMR was more appropriate for our purposes.

The discussion about discharge and reappraisal warrants more detail as well. Refer to the literature on coping styles and their relative effectiveness. Some quite deep topics are covered very superficially here and with insufficient reference to current research. It is unclear why the topic has suddenly shifted to that of listening to sad music.

We have clarified that anxious individuals who are using the discharge strategy (listening to music that reflects one’s current state) might listen to sad or angry music at line 79.

A quick scan in Google Scholar has revealed numerous papers that have already been published on the topic of music and COVID-19, none of which have been discussed here. What does the current study add to the knowledge already shared in these studies?

In the introduction, we now include significant findings from other studies with similar topics (100-111) and clarify how our study fits into this emerging literature. While some of our research questions are similar to what other researchers have published since the beginning of the pandemic, we don’t see this as a major weakness: the early stages of COVID-19 provided an unparalleled, and potentially once in a lifetime experimental setting in which having complementary and possibly replicated findings can only strengthen the findings of any one study.

p. 5 – the hypotheses are non-specific and vague. Is the first hypothesis a group comparison?

The main hypothesis is not a group comparison between countries, as previous research has suggested the universality of music’s ability to increase wellbeing. Rather, data from multiple countries was used to assess the generalizability of our findings and uncover any possible mediating/confounding factors. Our main hypothesis is that across different cultures, individual differences in personal COVID severity would predict the use of music to emotionally regulate using any of the B-MMR strategies (i.e. regression analysis). We have clarified this in lines 129-141.

For the second hypothesis, which traits would be associated with which strategies and why? This has not been well set up in the introduction. The tests addressing the second hypothesis are less interesting in the context of this research because they do not seem to be specifically related to COVID-19 and therefore the gap in the literature that is being addressed here needs to be more clearly explained in the Introduction.

We have clarified all hypotheses at the end of the Introduction section and specified that our main hypothesis was regarding trait measures of empathy and music emotion regulation strategies (lines 129-141). This is justified in the introductory paragraphs.

Methods – More detail about the online platforms used to recruit participants is needed.

Please clarify whether ‘native English speakers’ were specifically recruited or whether it was sufficient that they be proficient English speakers. In Italy, at least, this might have considerably narrowed the population to mostly people who were not born in Italy.

We have added more detail about Prolific (lines 153-156) and CloudResearch (161-162). We have also clarified that participants were recruited specifically for their proficiency in English. (Lines 164-166). Specifically, for Prolific, only participants who self-reported that they were fluent in English were able to complete the survey. For the Indian population, recruited using CloudResearch, potential participants are run through CloudResearch’s standard English literacy and attention check. Only participants who pass the check are sent through to the survey.

p. 10 – For the reader it is easier if the type of analysis performed is stated at the beginning of reporting the result of each test rather than listed under separate sub-headings in the Methods section.

We have moved all analyses descriptions to the beginning of each result.

Also please state what the IVs and DVs are and label the groups. A general report of analysis techniques can be included here, although the information on analysis of auditory features etc is necessary at this point.

We have stated the IVs and DVs for each analysis (and introduced this denotation on lines 289-290), and have moved the auditory feature descriptions to line 239.

p. 12 – Analyses seem to be a multiple analyses for each hypothesis rather than a single hypothesis test. As such the results are difficult to follow and seem rather exploratory rather than confirmatory. I would suggest that the hypotheses and the analyses performed in order to test them be refined and focused in order to provide a clearer pattern of results.

In the revised manuscript, we have reorganized and clarified our hypotheses (and sub-hypotheses) in the introduction and refer back to these numbered hypotheses in the results section. We have also made it more clear which measures were used to test our hypotheses versus which were more exploratory in nature.
